# Herbal Medicine and Hepatocellular Carcinoma: Applications and Challenges

**DOI:** 10.1093/ecam/neq044

**Published:** 2011-02-20

**Authors:** Yan Li, Robert C. G. Martin

**Affiliations:** Division of Surgical Oncology, Department of Surgery, University of Louisville School of Medicine, Louisville, KY 40202, USA

## Abstract

Use of herbal medicine in the treatment of liver cancer has a long tradition. The compounds derived from the herb and herbal composites are of considerable interest among oncologists. In the past, certain herbal compounds and herbal composite formulas have been studied through *in vitro* and *in vivo* as an anti-hepatocellular carcinoma (HCC) agent, enhancing our knowledge about their biologic functions and targets. However there is a significant distinction between the herbal medicine and the herbal production even though both are the plant-based remedies used in the practice. In this article, for the sake of clarity, the effective herbal compounds and herbal composite formulas against HCC are discussed, with emphasizing the basic conceptions of herbal medicine in order to have a better understanding of the prevention and treatment of HCC by herbal active compounds and herbal composite formulas.

## 1. Introduction

### 1.1. Herbal Medicine

Generally, the description of herbal medicine is the use of medicinal herbs, preparation made from a plant or plants, to prevent and treat diseases and ailments or to promote health and healing. However, it is important to distinguish “herbal medicine" and “herbal production", which is often overlooked [1, 2]. There is a significant distinction between the herbal medicine and the herbal production, both are the plant-based remedies used in the practice. Herbal production is the conventional medicine with definite ingredient(s) and definite pharmacological effects when the “plant drug" is for medical use. Whereas, the use of herbs in herbal medicine divorced from the context of the so-called “scientific information" and thus not as strongly scientifically validated is a specific discipline of herbal medicine that provides the therapeutic understanding of the medicinal use of herbs [3, 4].

A good example of herbal medicine is “Chinese herbal medicine", one branch of traditional Chinese medicine which focuses on naturalism and holistic health that can be traced back as far as 2100 BC in ancient China [5]. In Chinese herbal medicine, the herbs are classified according to their properties. The use of specific herb(s) to treat diseases depends on the sign and symptom of patients. The herbalists believe that illness is an imbalance status of the body, and the herbs, based on their various characteristics which are in the accordance with the law of nature, can neutralize the sign and the symptom thereby keep an overall balanced status in patient's body [6]. Similar to Chinese herbal medicine, is the Ayurvedic alternative herbal medicine of India, the balance between *agni* (representing strength, health and innovation) and *ama* (representing weakness, disease and intoxication) is also emphasized. Therefore, herbal medicine is a preparation made from a plant or plants and used for any of such purposes, and the biological ingredients of herbal remedies are extracted from natural substances such plants, animal parts, shells, insects and even stones and minerals.

In general, the herbs used in herbal medicine often result from a combination of herbs with multiple ingredients, called “herbal composite formula", to ensure effective actions on multiple targets simultaneously. Contrary to Western medicine that prefers the analytical approach, most composite formulas in Chinese herbal medicine are empirically based and the principles underlining the composite formula are relatively simple; however, not as strongly defined and can vary among herbalists. A “food model" could be a good example to understand the principles consisting of the composite formula. Foods are complex and contain many different constituents. The furnished materials are used by body to nourish, support and reproduce its vital activities, while the wastes and some toxic materials are eliminated by the body itself. The rule of the composite formula and the dosage for an individual with specific signs and symptoms is also in the same way similar to the dosage of materials to treat marasmus and diet materials to treat obesity. Although the choice of herbs or “composite formula" for some specific signs and symptoms may represent in part a trend towards mysticism and thus highly variable, the fact is that some herbal drugs contain ingredients that specifically treat diseases. These biological ingredients extracted from natural substances result in multiple effective actions on different biological molecular targets.

Modern biomolecular science has contributed to the interpretation of these multiple effective actions of herbs, and some important properties such as anti-virus, anti-inflammatory and anticancer have been recognized. As more information is becoming available, some “herbal drugs" are identified for the effects against hepatocellular carcinomas (HCC). This aim of this review article is to present the current state of herbal medicine as a chemopreventitive and chemotherapeutic agent in HCC.

### 1.2. HCC and Herbal Medicine

HCC is the fifth most common malignancy worldwide, and with a continuously increasing incidence [7, 8]. Three curative methods are currently available: orthotopic liver transplantation (OLT), surgical resection and local destruction (LD). However, only few patients qualify for the “curative therapies" because these strategies depend largely on the extent and location of the tumor and the underlying liver disease such as cirrhosis. Despite these curative options, the recurrence rate may be as high as 50% at 2 years [9, 10]. Therefore, prevention of recurrent HCC after (or before) successful curative therapeutic interventions needs to be improved in order to make an impact on long-term survival of these patients. Many adjuvant treatments such as trans-arterial chemo-embolization (TACE), anti-viral treatments and immunotherapy have been used but never confirmed if positive [11–14]. Palliative treatments for HCC are indicated if there is no curative treatment option, four palliative treatments are transarterial chemo-embolization, systemic chemotherapy, interferon and hormonotherapy [15]. However, palliative therapy of patients with HCC remains challenging as HCC is highly resistant to systemic therapies. Importantly, the incidence still nearly equals the mortality rate and more than 80% of patients present with advanced disease [15]. The overall disappointing results of both curative therapies and palliative treatments in advanced HCC patients support the research for other more active and specific treatments to be administered alone or in combination with the current therapy.

Currently, few medical interventions have been thoroughly tested in HCC, in contrast with the many tested in other highly prevalent cancers, such as lung, breast and colorectal cancer [16, 17]. There is an urgent need for new active and well-tolerated treatments to improve survival among patients with advanced HCC (palliative setting) and to increase long lasting remission after curative treatments (adjuvant setting). Studies, especially in China, on the prevention and treatment using herbal medicine against HCC have been accumulated during the past decades. Herbal compounds could affect all phases of HCC, including initiation, promotion and progression [18, 19]. The active development of innovative therapeutic approaches and molecularly targeted agents using herbal medicine could offer an opportunity to study the agents in HCC and gives new hope for the future. Despite massive investigation and effort, no such herb-drug for HCC treatment has been licensed to date. Actually, herbs are regulated in the USA under Dietary Supplement Health and Education Act only as “dietary supplements", as opposed to the Chinese category “traditional Chinese medicine". Regarding the current use of herbs, many questions cannot be answered definitively by the available scientific data. In some instances enough research has not been performed, and in others the final end point of the research is flawed.

## 2. Methods

**Figure 1:**
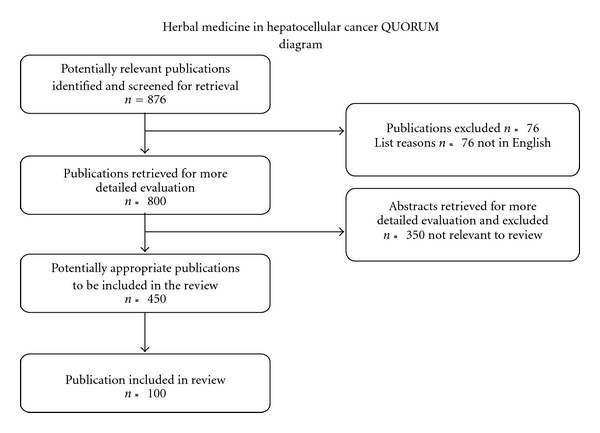
QUORUM algorithm of review of the herbal medicine publications and abstracts.

In this article, for the sake of clarity, we divided the herbal medicine into effective herbal compounds and herbal composite formula to discuss their anticancer properties against HCC. Several reviews have provided major contributions to the current knowledge on the herbal medicines for treatment of liver fibrosis and cancer [20–23]. The basic concepts of these “botanical drugs" are emphasized in order to have a better understanding of the prevention and treatment of HCC by herbal active compounds and herbal composite formula. The reports from both Chinese and English language are reviewed to provide a full picture of the progression of the use of herbal medicine against HCC. The English literature searches were conducted through Medline, Embase, Science Citation Index, Current Contents, PubMed databases, as well as relevant papers from integrative and complementary medicine journals, such as *Evidence Based Complementary and Alternative Medicine*, until January 2009. The outline of articles reviewed is presented in a Quality of Reporting of Meta-Analysis (QUORUM) flow chart showing the number of studies screened and included in the meta-analysis (Figure 1) [24]. Search items were “herb", “anticancer mechanism", “traditional Chinese medicine", “hepatoma", “hepatocellular carcinoma" and “hepatocellular adenocarcinoma". Restrictions were placed on language of publication, and only English was included. Studies lacking controls were excluded. Case reports were excluded. The relevant Chinese literature searches were carried out through Wanfang data until January 2009 using database of China Medical Association Journals (CMAJ), which is a portal to medical research materials published in China. The exclusion and inclusion for extracted data from literatures were same as that in PubMed. These restrictions were placed in order to have consistency among the reports reviewed. The aim of this review was to evaluate the current literature for the efficacy and safety of current herbal compounds in the treatment of hepatocellular cancer.

### 2.1. Herbal Compounds

**Figure 2:**
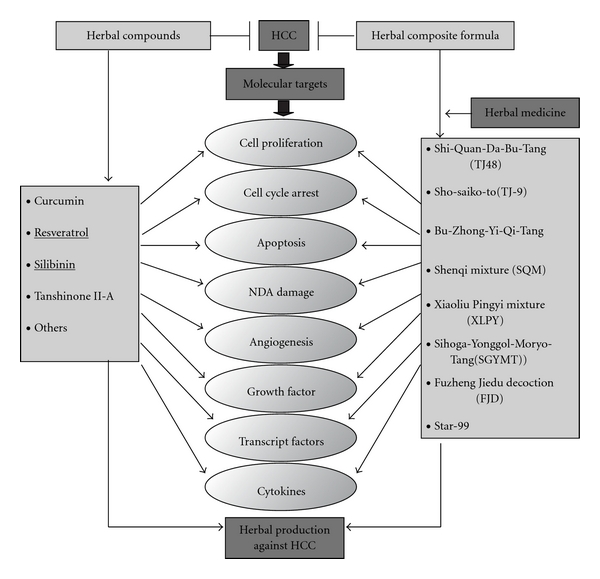
Summary results of the reported chemopreventitive effects of herbal compounds and herbal composite formulas.

There are a number of molecular compounds derived from the herbs that have been proven to be effective against HCC. Modern research is confirming that many compounds are active at some molecular targets which are being sought to find out potential newer generation “targeted" biological response modifier drugs [25, 26]. These herbal compounds have been shown to engage various molecular targets related HCC carcinogenesis and chemoprevention, which have been identified by laboratory research findings and clinical observations. These molecular compounds represent an enormous and almost untapped resource for HCC treatment (Figure 2). Some of the herbal compounds are discussed and summarized in Table 1.

#### 2.1.1. Curcumin

Curcumin (diferuloylmethane), a compound extracted from *Curcuma aromatica* widely used as a spice and coloring agent in food, possesses potent antioxidant, anti-inflammatory and anticarcinogenic properties. The anticarcinogenic property has been widely studied in various cancers [27]. Regarding HCC, three important properties of curcumin have been studied: anti-HCC; anti-angiogenesis of HCC; and anti-metastatic activity of HCC. Chuang et al. investigated the effect of curcumin on a HCC mouse model induced by *N*-diethylnitrosamine (DEN). They found that curcumin can inhibit effectively DEN-induced hepatocarcinogenesis in the C3H/HeN mice. The hepatic tissue from the DEN-treated mice showed a remarkable increase in the levels of p21(ras), expression of proliferating cell nuclear antigen (PCNA) and CDC2 proteins, while curcumin reversed the levels of all these biological markers. Another study performed by the same research group showed that curcumin can also suppress effectively DEN-induced liver inflammation and hyperplasia in a rat HCC model. Immunoblotting analysis showed that curcumin inhibits DEN-mediated the increased expression of oncogenic p21(ras), p53 proteins, PCNA, cyclin E, factor NF-*κ* and p34(cdc2), but not Cdk2, c-Jun and c-Fos [28, 29]. Yoysungnoen et al. evaluated the effect of curcumin and tetrahydrocurcumin on tumor angiogenesis of HCC mice. Human HCC cell line (HepG2) inoculated onto a dorsal skin-fold chamber of male BALB/c nude mice, and curcumin and tetrahydrocurcumin were fed oral daily at 300 and 3000 mg kg^−1^. The tumor microvasculature was observed using fluorescence videomicroscopy and capillary vascularity (CV) was measured. They found that treatment with curcumin and tetrahydrocurcumin resulted in significant decrease in the CV. The anti-angiogenic effects of curcumin and tetrahydrocurcumin were found to be dose-dependent. They concluded that the anti-angiogenic properties of curcumin and tetrahydrocurcumin represent a common potential mechanism for their anti-cancer actions [30–32].

Curcumin has also been shown to have potent anti-metastatic activity. Ohashi et al. analyzed the anti-metastatic mechanism using an orthotopic implantation HCC model with CBO140C12 cells. They found that daily oral administration of curcumin suppressed intrahepatic metastasis of orthotopic implanted HCC cells. They further examined the effect of curcumin on the metastatic properties *in vitro*, the results indicated that curcumin significantly inhibited adhesion and haptotactic migration to fibronectin and laminin thereby inhibiting tumor cells through Matrigel-coated filters [33]. An *in vitro* study carried out by Lin et al. also showed that curcumin could be a potential anti-metastatic agent against HCC, they found that curcumin, at 10 *μ*M, inhibited 17.4 and 70.6% of cellular migration and invasion of SK-Hep-1 cells, a highly invasive SK-Hep-1 cell line of human HCC. This anti-metastatic effect is associated with its inhibitory action on MMP-9 secretion [34]. There are also a number of studies for exploring the mechanism of curcumin against HCC. Lv et al. reported that curcumin can inhibit the level of histone deacetylase, enhance the expression of P21(WAF1/CIP1) protein and mRNA in HepG2 cells. They concluded that inhibiting histone deacetylase and increasing P21 may be one of the possible mechanisms of curcumin against HCC [35]. Cao et al. demonstrated that HepG2 cells treated with curcumin showed a transient elevation of the mitochondrial membrane potential, followed by cytochrome *c* release into the cytosol and disruption of DeltaPsim. Apoptosis was detected after curcumin treatment but the expression of Bcl-2 remained unchanged. They conclude that mitochondrial hyperpolarization is a prerequisite for curcumin-induced apoptosis in HepG2 cells [36, 37]. Chan et al. investigated the effect of curcumin on methylglyoxal-induced apoptotic events in HepG2 cells. In contrary, they report that curcumin significantly attenuates methylglyoxal-induced reactive oxygen species (ROS) formation thereby prevented cell apoptosis and apoptotic biochemical changes such as mitochondrial release of cytochrome *c*, caspase-3 activation, and cleavage of PARP (poly [ADP-ribose] polymerase) [38].

Although numerous *in vitro* and animal studies have shown that curcumin exhibits significant chemopreventitive effects and thus reported anti-HCC effect, the exact mechanism is largely unknown. The current reason for this lack of understanding comes from the fact that Curcumin is a complex herb made up of many potential active agents, *Curcuma aromatica*, *Curcuma longa* and curcumin oil to name a few, all of which have not been defined as to where the most active agent(s) are effective in chemoprevention. In addition, poor systemic absorption of curcumin is still a major obstacle for its application [39, 40].

#### 2.1.2. Resveratrol

Resveratrol, a polyphenol found in grape skins, peanuts, berries and red wine, has been shown to possess potent growth inhibitory effects against various human cancer cells including HCC. Resveratrol can be absorbed rapidly and accumulate in the liver. Lancon et al. studied the absorption and the efflux of resveratrol in the HepG2 cells. They found that resveratrol was rapidly conjugated and it entirely metabolized at 8 h to form two main resveratrol metabolites: monosulfate and disulfate [41]. The effect of resveratrol on HCC has been also extensively studied. Bishayee et al. evaluated the inhibitory effect of resveratrol against hepatocarcinogenesis using a two-stage HCC rat model. The HCC model was reproduced by a single intraperitoneal injection of diethylnitrosamine (DENA), followed by promotion with phenobarbital in drinking water. They found that resveratrol exerts a significant chemopreventive effect on DENA-initiated hepatocarcinogenesis through inhibition of cell proliferation and induction of apoptosis. They concluded the possible mechanism could be that the resveratrol-induced apoptogenic signal is mediated through the downregulation of Bcl-2 and upregulation of Bax expression [42]. An *in vitro* study carried out by Stervbo et al. also showed that the inhibitory effects of resveratrol on cell proliferation and apoptosis in the HepG2 cells. They found that resveratrol inhibited DNA synthesis and increased nuclear size and granularity at G1 and S phases of HepG2 cells. Apoptosis was also stimulated by resveratrol in a concentration-dependent manner in HepG2 cells. They concluded that resveratrol inhibits cell proliferation by interfering with different stages of the cell cycle, and causes stimulation of apoptosis [43]. Notas et al. also used the HepG2 cells to address the action of resveratrol on cell growth and to examine some possible mechanisms. Their results indicate that the stilbene resveratrol inhibits cell proliferation, reduces the production of ROS and induces apoptosis, through cell-cycle arrest in G1 and G2/M phases. They also found that stilbene resveratrol modulates the NO/NOS system, by increasing iNOS and eNOS expression, NOS activity and NO production [44]. Yan et al. investigated the effects of resveratrol on proliferation and gap-junctional intercellular communication (GJIC) in HepG2 cells. They found that resveratrol arrests HepG2 cell growth in S phase, inhibits DNA synthesis and induces cell apoptosis. The levels of GJIC increased sharply after resveratrol treatment implied that the increased GJIC level could play a role on the effect of resveratrol in the cancer chemopreventive activity [45]. The study carried out by Ma et al. agreed with the observations above. They further investigated the effects of resveratrol on mitochondrial membrane potential and cell morphology of HepG2 cells. Their results showed that resveratrol at high concentrations can obviously cause sharp increment of the mitochondria membrane potential. They concluded that the capacity of resveratrol for inhibiting proliferation and inducing cell apoptosis could be resulted in depolarizing mitochondrial membrane potential [46].

Like curcumin, potent anti-metastatic activity of resveratrol has been also investigated. Yu et al. investigated the effects of resveratrol on invasion ability of human HCC cells and tumor necrosis factor-alpha (TNF-*α*)-mediated MMP-9 expression. They found that resveratrol significantly inhibited TNF-*α*-mediated MMP-9 expression, NF-kappa B expression and invasion in HepG2 cells. They concluded that the inhibition of TNF-*α*-mediated MMP-9 expression and the potential invasion by resveratrol are partly associated with the downregulation of the NF-kappa B signaling pathway [47]. Studies carried out by Miura et al. demonstrated that dietary resveratrol can inhibit metastasis of hepatoma in Donryu rats subcutaneously implanted with an ascites hepatoma cell line of AH109A. They found that ROS accelerated the invasive capacity of a rat ascites hepatoma cells, and resveratrol suppressed the ROS-potentiated invasion of the hepatoma cells [48, 49]. Despite the encouraging achievement regarding the anti-HCC effects of resveratrol, the low bioavailability and the potential toxicity by the modulation of liver genes at the high dose are also observed [50, 51]. Again, as with Curcumin the specific active agents in resveratrol have not been accurately defined, and clinical trial researchers have been plagued by the lack of systemic absorption that can be achieved through oral administration.

#### 2.1.3. Silibinin

Silibinin, a polyphenolic flavonoid, is the major biologically active compound of milk thistle. It is well known that milk thistle is safe and well-tolerated, and it protects the liver from drug or alcohol-related injury [52, 53]. Silibinin and its crude form, silymarin, are used clinically and as dietary supplements against liver toxicity. A randomized controlled multicenter trial has shown that daily administration of silymarin for several years results in a significant reduction in the mortality of patients suffering from alcoholic liver cirrhosis [54]. Studies have demonstrated the inhibitory effects of silibinin on multiple cancer cell lines including HCC [55, 56]. Varghese et al. investigated the effects of silibinin on cell growth, cytotoxicity, apoptosis and cell cycle in two different HCC cell lines, HepG2 (hepatitis B virus negative; p53 intact) and Hep3B (hepatitis B virus positive; p53 mutated). They found that silibinin strongly inhibited growth of both HepG2 and Hep3B cells. Silibinin also caused G1 arrest in HepG2, and G1 and G2-M arrests in Hep3B cells. Further studies showed that silibinin induces Kip1/p27 but decreases cyclin D1, cyclin D3, cyclin E, cyclin-dependent kinase (CDK)-2, and CDK4 levels in these two cell lines. In Hep3B cells, silibinin also reduced the protein levels of G2-M regulators. CDK2, CDK4, and CDC2 kinase activity were strongly inhibited in these HCC cells by silibinin [57]. Lah et al. investigated the effect of silibinin on HCC cell growth in four human HCC cell lines: HuH7, HepG2, PLC/PRF/5 and Hep3B cells. After treated with different doses of silibinin, proliferation, apoptosis, cell-cycle progression, histone acetylation and other related signal transductions were examined. They demonstrated that silibinin significantly inhibited the growth of HuH7, HepG2, Hep3B and PLC/PRF/5 human hepatoma cells. In addition, they also found downregulated levels of metalloproteinase-2 (MMP2) and CD34 in the HCC cells, which could be a possible anti-angiogenic mechanism of silibinin. They also demonstrated that silibinin increased acetylation of histone H3 and H4 (AC-H3 and AC-H4), indicating a possible role of altered histone acetylation in chemoprevention of silibinin against HCC cells [58]. Momeny et al. evaluated the effect of silibinin on HepG-2 cells regarding the biomarkers of cell proliferation, cytotoxicity, metastatic potential, nitric oxide (NO) production, ERK 1/2 phosphorylation and activation in HepG-2 cells. They found that silibinin inhibited cell proliferation, matrix MMP-2 enzymatic activity, NO production and ERK 1/2 phosphorylation without exerting any cytotoxicity effect. The possible mechanism of silibinin against HCC could be inhibiting cell proliferation and invasive potential of HepG-2 cells through inhibition of ERK 1/2 cascade [59].

Currently, silibinin has not been evaluated for human effects or toxicity in human clinical trials and thus only holds potential promise as an active chemopreventitive agent.

#### 2.1.4. Tanshinone II-A

Tanshinone IIA, one of the most abundant diterpenes isolated from *Salvia miltiorrhiza Bunge* (Danshen in Chinese). Tanshinone IIA has been shown to possess pharmacological activities including antioxidant [60], protecting and/or preventing angina pectoris and myocardial infarction [61]. Report has shown that inhibition of proliferation and cytotoxic effects on cell lines derived from various human carcinomas [62, 63]. Yuan et al. evaluated the effects of tanshinone II-A on growth inhibition and apoptosis of human HCC cells (cell line SMMC-7721). The growth and colony-forming of SMMC-7721 cells were obviously suppressed after tanshinone II-A treatment. The apoptosis index was significantly increased and the cells were arrested in G(0)/G (1) phase. In addition, expressions of apoptosis-related genes bcl-2 and c-myc were downregulated, while fas, bax, p53 upregulated [64]. Zhong et al. investigated the effect of tanshinone IIA on the growth and apoptosis in HepG2 cells. They found that tanshinone IIA not only inhibited the cell growth, but also induced apoptosis in HepG2 cells [65]. Tang et al. studies the effect of tanshinone IIA on growth and apoptosis in human HCC cell line BEL-7402. Growth suppression and induced cell apoptosis were found as BEL-7402 cells treated with tanshinone IIA [66]. Wang et al. evaluated the proliferation of human HCC cell line (SMMC-7721) treated with tanshinone by Brdu labeling and PCNA immunohistochemical staining. They found decreased indexes of Brdu labeling and PCNA detection after tanshinone treatment. The inhibitory effect of tanshinone on cancer cell growth might associate with inhibiting DNA synthesis [67, 68]. Li et al. performed *in vitro* and *in vivo* studies using polylactic acid nanoparticles containing tanshinone IIA (TS-PLA-NPs) against HCC. They found that tanshinone IIA in TS-PLA-NPs were effective in destroying the human liver cancer cells. Tanshinone IIA in TS-PLA-NPs also prevented tumor growth and increased survival rate of mice with hepatoma [69].

#### 2.1.5. Other Reported Compounds

Beside these widely investigated compounds, some other potential compounds for the chemopreventive effect against HCC have been also evaluated. Salvianolic acid B is a major water-soluble polyphenolic acid extracted from *Radix Salviae miltiorrhizae* (Sm). Studies have shown that Salvianolic acid B can improve acute and chronic liver, decrease the serum alanine aminotransferase (ALT) and aspartate aminotransferase (AST) levels and enhance the total prostaglandin content in liver mesenchymal cells in injured rats [70]. Baicalein is a flavonoid from baikal skullcap root. Matsuzaki et al. investigated the anti- HCC effect of baicalein. They found that treatment with baicalein strongly inhibited the activity of topoisomerase II, induced apoptosis and suppressed the proliferation the HCC cell lines [71]. Pheophorbide a (Pa) is an active compound from *Scutellaria barbata*. Tang et al. used a multi-drug resistant (MDR) HCC cell line (R-HepG2) to evaluate the anti-proliferative effect of Pa. They found that Pa can significantly inhibit the growth of R-HepG2 cells [72]. It has been showed that Pa can enhance the efficacy of photodynamic therapy (PDT) for HCC, very likely due to its antiproliferative and pro-apoptotic effect [73–75].

### 2.2. Herbal Composite Formula

Besides these promising molecule compounds isolated or derived from herbals, there is a number of herbal complex formulas prescribed by Chinese medicine practitioners defined as either a traditional medical doctor or an herbal pharmacists for treating and preventing HCC. Using state-of-the-art technology, the techniques of herbal extraction and purification have been dramatically improved and innovated in the Chinese herbal medicine industry. The traditional forms of herbal preparation such as decoction, powder, plasters and pill have been developed into the modern forms, capsules, tablets and injection which as more convenient and palatable. The technological improvement of herbal preparation also made it becoming possible for better control the constituents of the herbal composite formula.

Although some herbal composite formula showed efficacy against HCC, it is worth mentioning that the usage of herbal composite formula is from the herbal medicine practitioner's point of view which is quite different from that of conventional Western medicine, that is, not validated in a standard Phase 1, Phase 2 or Phase 3 clinical trials. When a formula is composed using different herbs, from the herbal medicine principle, the whole unhealthy condition should be treated to make body homeostasis other than to “cure" tumor only. Generally, herbal composite formula can be divided into two categories: classic composite formula and experience composite formula. Classic composite formula is an ancient formula with definite constituent of herbs. Classic composite formula can be traced back as far as thousands years, while experience composite formula is a contemporary composite formula which is generally modified from classic composite formula. Experience composite formula is usually not with definite constituent of herbs, and the constituent of composite formula can be changed with addition or deletion of herbs according to the symptoms of patients. Both classic composite formula and experience composite formula are discussed and summarized in Table 2.

#### 2.2.1. Clinical Studies of Classic Herbal Composite Formula

Shi-Quan-Da-Bu-Tang (TJ-48, Juzen-taiho-to in Japanese,) is a classic herbal composite formula. It consist of 10 herbs; Mongolian milkvetch root, Cinnamon, ginseng root, largehead atractylodes rhizome, tuckahoe, liquorice root, Chinese angelica, radix rehmanniae root, white peony root, szechwan lovge rhizome. TJ-48 has been used extensively in medical practice in Asia even though their mechanism of action remains elusive. Tsuchiya et al. studied the protective effect of TJ-48 against hepatocarcinogenesis in HCC patients after surgical treatment. A significantly longer intrahepatic recurrence-free survival was observed in the TJ-48 group even though most of the patients experienced recurrence of HCC [87].

Xiao-Chai-Hu-Tang (TJ-9, Sho-saiko-to in Japanese), a classic herbal composite formula, is commonly administered to patients with chronic viral liver disease in order to improve their overall physical condition and to prevent the development of liver cancer. The crude extracts of TJ-9 consist of seven herbs: bupleurum root, pinellia tuber, scutellaria root, jujube fruit, ginseng root, glycyrrhiza root and ginger rhizome. A prospective, randomized, non-blind controlled study was carried out by Oka et al. to evaluate the preventive effect of TJ-9 on HCC development. Two hundred and sixty patients with cirrhosis were randomly assigned to two groups, matched for age, sex, presence of hepatitis B surface antigen and the severity of liver damage. The patients in the trial group were given TJ-9 at a daily oral dose of 7.5 g in addition to the conventional drugs given to the control patients, and prospectively monitored for 60 months. They found that the survival curve for 5 years of the trial group without Hepatitis B antigen was significantly higher than that of the control group. They concluded that TJ-9 could help to prevent the development of HCC in patients with cirrhosis, particularly in patients with negative hepatitis B antigen [76]. The flaw in this study was that the hepatitis B antigen patients were a subset analysis and the study was underpowered and at risk for a type 1 error in it efficacy with hepatitis B patients. A study from the same group further evaluated the potential of TJ-9 against HCC in pairs of patients matched for age, sex, presence of hepatitis B antigen and the scores of the severity of liver dysfunction from 260 cirrhotic subjects. They found the incidence of HCC was significantly lower in the trial group, 17 HCC patients in the control versus 9 HCC patients in the trial group. They concluded that TJ-9 may prevent or delay the emergence of latent HCC in patients with cirrhosis [88]. The limitation of this study is the concern for duplication of patients studied. These results came from the same authors, over the same time interval, and there is no mention that these two groups of patients were different. Thus while TJ-9 may hold promise, further regulated studies are needed beyond a single institutional study to validate this initial encouraging results.

Bu-Zhong-Yi-Qi-Tang (BZYQT) is a classic herbal composite formula in Chinese herbal medicine, it consist of eight herbs; Mongolian milkvetch root, liquorice root, largehead atractylodes rhizome, ginseng root, Chinese angelica, skunk bugbane rhizome, bupleurum root and tangerine. Kao et al. investigated the effects of BZYQT on granulocyte colony-stimulating-factor (G-CSF) and TNF-*α* production by peripheral blood mononuclear cells (PBMC) in healthy volunteers and HCC patients. They found that the productions of G-CSF and TNF-*α* in PBMC of both volunteers and HCC patients were significantly stimulated by BZYQT. However, the increments of G-CSF and TNF-*α* in HCC patients are not as high as in healthy volunteers. They suggested that BZYQT is a unique formula for the stimulation of PBMC to produce G-CSF and TNF-*α*, and administration with BZYQT may be beneficial for increasing defensive mechanism in patients against HCC [81]. The limitations of this study are the lack of pharmacokinetic distribution of BZYQT effects in HCC patients, which may be its major limitations in a larger clinical evaluation.

#### 2.2.2. Clinical Studies of Experience Herbal Composite Formula

Because the focus of composing a formula is not in the direct “killing" of tumor cells, herbal composite formula is usually considered as palliative treatment and adjuvant treatment for HCC. The purpose for clinical use of experience herbal composite formula is to recover liver function, alleviate symptoms and to improve life quality. Most studies of use of experience herbal composite formula are retrospective, and the constituent of herbs in composite formula is not well defined. The experience herbal composite formula usually combines with the conventional palliative treatment such as transcatheter arterial chemoembolization (TACE) and systemic chemotherapy (Table 3).

A retrospective study was undertaken by Chen et al. to evaluate the effect of a complex prescription of Chinese crude drug on the hepatic function and some symptoms in HCC patients post-TACE. Forty-five HCC patients post-TACE were treated with a complex prescription of herbal crude, other 37 patients as the control group treated by routine therapy. The symptoms such as anorexia, nausea, abdominal distention and lassitude along with liver function and *α*-fetoprotein (AFP) were observed. At the end of the therapy, both symptoms and hepatic function were improved. They concluded that the complex prescription of Chinese crude drug could benefit for HCC patients post-TACE by the recovery of liver function and improvement of the life quality [91].

Meng et al. performed meta-analysis to compare the efficacy and safety of complex prescription of Chinese crude drug plus TACE (therapy I) with that of TACE alone (therapy II). In total, in 37 trials involving 2653 patients, the results showed that therapy I, compared with therapy II, improved patient survival, quality of life, alleviated symptoms and increased tumor response. Combining of complex prescription of Chinese crude drug with TACE are thus more therapeutically beneficial. No serious adverse events were reported in therapy I group [89].

Shu et al. evaluated the effectiveness of Chinese herbal medicine combined with chemotherapy. They searched the databases TCMLARS, PubMed and EMBASE as well as the bibliographies of studies identified in the systematic search for potentially relevant titles or abstracts of studies in any language. They applied random effects meta-analysis in 26 studies representing 2079 patients met the inclusion criteria. They found that Chinese herbal medicine combined with chemotherapy improved survival rate and tumor response compared with chemotherapy alone [90].

Shenqi mixture (SQM) is an herbal composite formula from Ginseng root and Mongolian milkvetch root. Lin et al. investigated the short-term efficacy and safety of SQM combined with microwave coagulation in treating primary HCC. Tumor size, patients' symptoms, serum level of AFP, and some immune parameters were evaluated. The results indicated that SQM decreased tumor size and AFP. The levels of CD3^+^, CD4^+^, CD4^+^/CD8^+^, NK activity, Karnofsky scores and hepatic function were improved with SQM plus microwave coagulation treatment compared to those in the microwave coagulation alone. SQM also improved the survival rate and symptoms such as hepatic region pain, fever, weakness, poor appetite and jaundice [82].

#### 2.2.3. Experimental Studies of Herbal Composite Formula

**Cell Culture Studies**

The *in vitro* anti-HCC activity of herbal composite formula with either indefinite constituent or definite constituent has been studied widely. Chao et al. investigated the effect of the extracts from *Lycium barbarum* (LBE) and *Rehmannia glutinosa* (RGE) on the cell proliferation and apoptosis in both rat (H-4-II-E) and human (HA22T/VGH) HCC cells. They incubated these two HCC cell lines with various concentrations of LBE and RGE, and measured the cell proliferation, apoptosis and p53 protein. They found that both LBE and RGE inhibited proliferation of H-4-II-E cells and of HA22T/VGH cells. Apoptosis and p53 significantly were increased in H-4-II-E cells treatment with crude LBE and RGE [92]. Chang et al. studied the chemopreventive potential of the extract of *Cornus officinalis Sieb. et Zuce* against HCC using three HCC cell lines (HepG2, SK-Hep1 and PLC/PRF/5). We discuss *C. officinalis Sieb. et Zuce* as herbal composite formula for the following two reasons. First, according to Chinese herbal medicine, sometimes single herb can be a composite formula, that is, Ginseng Decoction is content of only ginseng. Secondly, *C. officinalis Sieb. et Zuce* is not a single compound. There could be hundreds compounds in the extracts of *C. officinalis Sieb. et Zuce.* Chang et al. found that extract of *C. officinalis Sieb. et Zuce* inhibited growth in all these HCC cells. They concluded that *C. officinalis Sieb. et Zuce* might be a candidate for chemopreventive agent against HCC through anti-neoplastic effects [93]. Li et al. investigated Xiaoliu Pingyi Mixture (XLPY) induced apoptosis in human HCC cell line H-7402. Instead of directly adding herbal agent into culture media, they used serum pharmacologic method, serum loaded with herbal ingredients to treat cells. They found that XLPY loaded serum can significantly inhibit HCC cell growth and induce apoptosis. Bcl-2 gene expression was also inhibited in H-7402 cells treated with XLPY [83].

TJ-9, as a typical classic composite formula, has been investigated regarding its anti-HCC effect. The ingredients from this classic composite formula have been also evaluated widely to explore more efficient l compounds against HCC. Yamashiki et al. investigated the effect of TJ-9 on TNF-*α* and G-CSF in peripheral mononuclear cells of patients with HCC accompanied by liver cirrhosis. They found that TJ-9 can increase the levels of TNF-*α* and G-CSF. They concluded that TJ-9 could enhance the biological defense mechanism through increasing TNF-*α* and G-CSF thereby improving the overall physical condition in HCC patients [94]. Okita et al. investigated the ingredients of TJ-9 (baicalein, baicalin, saikosaponin-a, saikosaponin-c, ginsenoside Rb1, ginsenoside Rg1) on cultured human hepatoma cells (HuH-7). Cell-cycle analysis was carried out with flow cytometry and the bromodeoxyuridine (BrdU)-labeling method. They found that baicalein, baicalin and saikosaponin-a inhibited cell proliferation dose-dependently. In addition, saikosaponin-a possesses a strong cell-killing effect, however, saikosaponin-c, ginsenoside Rb1 and ginsenoside Rg1 had no effect on cell proliferation [77]. Yano et al. compared the individual ingredients (saikosaponin a, c and d, ginsenoside Rb1 and Rg1, glycyrrhizin, baicalin, baicalein and wogonin) from TJ-9 to TJ-9 composite formula using a human HCC cell line (KIM-1). They found that TJ-9 composite formula suppressed significantly the proliferation of KIM-1cells compared with any individual ingredient from TJ-9 composite formula. TJ-9 composite formula reduced the number of preneoplastic cells [78].

Chai-Hu-Jia-Long-Gu-Mu-Li-Tang (SGYMT; Saiko-ka-ryukotsu-borei-to in Japanese; Sihoga-Yonggol-Moryo-Tang in Korean) is a classic composite formula. The crude extracts of SGYMT consist of 11 herbs: bupleurum root, dragon's bone, rhubarb, pinellia tuber, scutellaria root, cassia twig, rhubarb, jujube fruit, ginseng root, glycyrrhiza root and ginger rhizome. Ha et al. studied the extracts prepared from SGYMT and its herbal ingredients for the inhibitory effects on tumor-specific matrix metalloproteinases-2 and -9 (MMP-2/9) activities in HCC cell line (SK-Hep1 cells). They found that SGYMT decreased the activities of MMP-2 and -9. However, the cytotoxicities of SGYMT and its ingredients on SK-Hep1 cells were very low. The inhibitory effect on the invasion of SK-Hep1 cells using matrigel precoated transwell chambers showed that SGYMT effectively inhibited the invasion of SK-Hep1 cells as compared to the control groups. They concluded that SGYMT could be used as potential anti-tumor metastasis agent [79].

**Animal Model Studies**

QHF is an herbal composite formula including three category herbs (according to Chinese herbal principle), in which Q (Qingrejiedu) represents “clean up pathogenic and toxic materials"; H (Huoxuehuayu) represents “improve micro blood circulation"; and F (Fuzhengguben) represents “enhance defending capability". QHF consist of mainly by Ginsenosides Rg3, Lentinan, Tanshinone and Norcantharidin. Chen et al. investigated the active ingredients in QHF formula against HCC. They observed the anti-HCC effect of QHF combined with cisplatin in a HCC cells implanted mouse model. The results indicated that treatment with the QHF formula was efficient not only in inhibiting the growth of HCC cells, but also in prolonging the life of the HCC mice. In addition, QHF combined with cisplatin can ameliorate cisplatin-induced leucopenia, spleen and thymus atrophy and other toxic reactions [84].

Yin et al. investigated the effect of Fuzheng Jiedu Decoction (FJD), an experience composite formula, against HCC using male BALB/c athymic mouse model. They observed survival rate, volume of tumors, and intrahepatic metastasis. The results indicated that FJD significantly increased the total survival rate and decreased tumor intrahepatic metastasis. Immunohistochemistry showed that an increased intensity of phosphatase and tensin homolog deleted on chromosome ten (PTEN) staining in tumor tissue treated with FJD. They concluded that FJD can prolong the survival and decrease tumor intrahepatic metastasis. The possible mechanisms of FJD against HCC could be through enhancing the expression of PTEN in the compromised liver [85].

Lin et al. investigated the anti-cancer effect by tumor focal injection of Chinese herbal composite formula Star-99 in a HCC mouse model. Nude mice transplanted with human HCC SMMC-7721 cells were intratumorally injected with Star-99 every 5 days with a total of four injections. Tumor value, growth index and apoptosis were evaluated. They found that Star-99 can decrease the tumor size, and increase apoptotic index significantly. They concluded that Star-99 markedly destructs HCC cells *in vivo* and it is feasible in the treatment of HCC [86].

Qian et al. used *Bletilla striata* as TACE agent to treat HCC rats. *Bletilla striata* was milled into a micro-particle (diameter 45 *μ*m), and rat HCC model was induced by hepatic implantation with hepatoma cells. They found that application of *B. striata* combined with mitomycin using TACE inhibited the growth of tumor which implanted in the liver. Importantly, the survival rate of HCC rats was also significantly improved. They concluded that *B. striata* could be a valuable anti-HCC as a TACE agent [95].

There are some *in vivo* studies regarding the classic herbal composite formula. Tsuchiya et al. performed a DEN induced HCC mouse model to evaluate the effect of TJ-48 on HCC. They found that TJ-48 inhibited the development of liver tumors along with the reduced oxidative DNA damage, inflammatory cell infiltration and cytokine expression [87]. Shiota et al. investigated the preventive effect of TJ-9 on hepatocarcinogenesis using a DEN-induced HCC rat model. They found that TJ-9 reduced the number of preneoplastic cells, detected as the glutathione S transferase P (GST-P)-positive hepatocytes, inhibited the development of liver tumors, and significantly decreased the formation of 8-hydroxy-2′-deoxyguanosine (8-OHdG). They concluded that TJ-9 prevents hepatocarcinogenesis in association with inhibition of 8-OHdG formation [96]. Although TJ-9 has been showed encouraging effect for anti-HCC in most studied in clinical patient and cell culture, some disagreement also exist. Watanabe et al. investigated the outcome of administration of TJ-9 on the occurrence of hepatic neoplasia in Long-Evans Cinnamon rats, a spontaneous HCC model. TJ-9 was administered from 6 weeks of age until the rats were sacrificed at 76 weeks, at which time most of the non-treated animals were known to have HCC. However, in regard of HCC, the percent area, number of areas, and mean size of area staining positively for GST-P revealed no significant differences between the groups. The number of GST-P-positive areas within the HCC lesions was even greater in the TJ-9 group than in the control. They concluded that long-term administration of TJ-9 did not reduce the risk of hepatocarcinogenesis in LEC rats [80].

## 3. Future Study and Safety

Through centuries of clinical practices in herbal medicine, there could be a number of candidate drugs derived from the herbs or herbal composites formulae for chemoprevention and chemotherapeutic strategy against HCC. Certain herbal compounds and herbal composite formula have been studied through *in vitro* and *in vivo* as an anti-HCC agent for many years, enhancing our knowledge about their biologic functions and targets. Despite studies for decades as well as advancement of our understanding of the molecular targets, there are still some concerns of herbal medicine. The first is the use of composite formula (usually mixed extracts with uncertain ingredients) could result in dramatically different outcome for clinical trials and experimental studies. To address this issue, the biologically active substances in herbs need to be defined and standardized. Simplifying the herbal compounds to several biologically active compounds could be a way to standardized clinical trails and experimental studies. However, the biologically active effects of herbal composite formulas could be from the interactions between herbs and compounds, and one should be cautious to avoid eliminating the compound(s) within from the herbal composite formula even though the compound(s) may not have any biologically active effect by themselves (itself). The second issue is the relationship between clinical studies and experiments in test tubes and in animals. Many clinical trials in China focusing on the anticancer effects of herbal composite formulas have been conducted. There is a lack of randomized, placebo-controlled clinical studies regarding the use of both herbal compounds and composite formula. High-quality, rigorously controlled clinical trials are needed to avoid the overstated effect of herbal compounds and composite formula. On the other hand, these clinical trials provided some clues to further investigate the pharmacological effects of the herbal compounds and composite formula being used “effective" against HCC, which only can be demonstrated definitively by the scientific data. Thus future clinical trials should evaluate a dedicated defined reproducible herbal compound to allow for standardizing therapy and efficacy.

The third is concern about the safety of herbal remedies. Traditional medical doctor, or herbal pharmacists recommend the traditional forms of herbs that are organic, unadulterated, unprocessed and prepared in traditional ways, just like any dietary ingredient, the processing methodologies are described in Chinese, Japanese, Korean Pharmacopoeias. They simply trust the wisdom of the body to recognize and make use of herbs. However, the reports of the herbal toxic effects contradict the popular view that herbals are natural and harmless. Among the herbal toxic effects, hepatotoxicity is the most frequent. The investigation of compounds and composite formula regarding safety and toxicity is needed before definitive clinical guidelines can be made.

Thus in summary herbal compounds have been shown to be efficacious and safe in small single center studies in the treatment and prevention of HCC and cirrhosis. However, on going standardization of the preparation, purity, and active compounds in conjunction with natural constituents should be specified for quality control aspects and must be established in order for future Phase 2 and Phase 3 trials can be created to verify these intriguing and optimistic results in this devastating disease.

## Figures and Tables

**Table 1:** Summary of anti-HCC herbal compounds.

Compounds	Possible mechanisms of anti-HCC	References
Curcumin	Inhibits proliferation; induces apoptosis; inhibits p21(ras), PCNA, cyclin E, factor NF-*κ* and p34(cdc2); anti-angiogenesis; inhibits MMP-9 secretion; inhibits histone deacetylase; enhances P21(WAF1/CIP1) protein; elevates mitochondrial membrane potential; attenuates ROS	[27–40]
Resveratrol	Inhibits proliferation; induces apoptosis; downregulates Bcl-2 and upregulates Bax expression; reduces ROS; induces cell-cycle arrest in G1 and G2/M phases; modulates NO/NOS; increases gap-junctional intercellular communication; sharps increment of the mitochondria membrane potential; inhibits TNF-alpha-mediated MMP-9 expression; suppresses the ROS-potentiated invasion.	[41–51]
Silibinin	Causes G1 arrest; induces Kip1/p27; decreases cyclin D1, cyclin D3, cyclin E, cyclin-dependent kinase (CDK)-2, and CDK4; downregulates metalloproteinase-2; increases acetylation of histone H3 and H4; inhibits cell proliferation; inhibits NO production and of ERK 1/2 cascade.	[52–59]
Tanshinone IIA	Induces apoptosis; induces cell arrested in G(0)/G(1); downregulates bcl-2 and c-myc; upregulates fas, bax, p53; inhibits DNA synthesis.	[60–69]

**Table 2:** Summary of anti-HCC herbal composite formula.

Herbal composite formula	Possible mechanisms of anti-HCC	References
Shi-Quan-Da-Bu-Tang (TJ-48)	Inhibits tumors growth, reduces oxidative DNA damage, inflammatory cell infiltration and cytokine expression	[76]
Sho-saiko-to (TJ-9)	Increases TNF-*α* and G-CSF; inhibits 8-OHdG formation; reduces the number of pre-neoplastic cells; cell-killing effect	[77–80]
Bu-Zhong-Yi-Qi-Tang	Stimulates productions of G-CSF and TNF-*α*	[81]
Shenqi mixture (SQM)	Increases CD3^+^, CD4^+^, CD4^+^/CD8^+^, NK activity	[82]
Xiaoliu Pingyi Mixture (XLPY)	Inhibits growth; induces apoptosis; inhibits Bcl-2 gene	[83]
Sihoga-Yonggol-Moryo-Tang (SGYMT)	Inhibits MMP-2 and MMP-9; inhibits tumor invasion	[79]
QHF	Inhibits the growth of HCC	[84]
Fuzheng Jiedu Decoction (FJD)	Enhances PTEN; inhibits tumor invasion	[85]
Star-99	Inhibits the growth of HCC; induces apoptosis	[86]

**Table 3:** Summary of clinical reports using herbal medicines to treat patients with HCC.

Classic herbal composite formula	Patient numbers (treated/control)	Dosing and duration	The anti-HCC effects	Refs
Shi-Quan-Da-Bu-Tang (TJ-48)	48 (10/38)	7.5 g daily oral dose. For up to 6 years	Inhibiting tumors growth. Improving intrahepatic recurrence-free survival after surgical treatment of HCC	[76]
Sho-saiko-to (TJ-9)	260 (130/130)	7.5 g daily oral dose. For up to 2.5 years	Preventing the development of HCC in patients with cirrhosis, particularly in patients with negative HBs antigen	[77, 79]
Shenqi mixture (SQM) combined with microwave coagulation	72 (36/36)	20 ml, three times a day for 1 month	Improving clinical symptoms and the quality of life. Prolong the survival period of patients	[82]
*Experience herbal composite formula*				
Complex prescription of Chinese crude drug after TACE therapy	82 (45/37)	Dosing unavailable. Duration 1 month	Reducing the side effect of TACE. Improving liver function and life quality	[89]
Complex prescription of Chinese crude drug combined with TACE therapy	2653 (unavailable/unavailable)	Dosing and duration unavailable	Improving patient survival and life quality. Alleviating symptoms. Increasing tumor response	[82]
Complex prescription of Chinese crude drug combined with chemotherapy	2079 (unavailable/unavailable)	Dosing and duration unavailable	Improving survival rate and tumor response	[90]

## References

[1] Weiss R (1988). *What is Herbal Medicine*.

[2] Liu G (2001). *Chinese Herbal Medicine (Clinical Essentials Series*.

[3] Liu G (2002). *Development of Formulas of Chinese Medicine*.

[4] Liu G (2002). *Fundamentals of Formulas of Chinese Medicine*.

[5] Yang L (2009). *Book of Changes And Traditional Chinese Medicine*.

[6] Cheng H (1995). *Advanced Textbook on Traditional Chinese Medicine and Pharmacology*.

[7] Fattovich G, Stroffolini T, Zagni I, Donato F (2004). Hepatocellular carcinoma in cirrhosis: incidence and risk factors. *Gastroenterology*.

[8] Llovet JM (2005). Updated treatment approach to hepatocellular carcinoma. *Journal of Gastroenterology*.

[9] Nagasue N, Kohno H, Chang Y-C (1993). Liver resection for hepatocellular carcinoma: results of 229 consecutive patients during 11 years. *Annals of Surgery*.

[10] Yamamoto J, Kosuge T, Takayama T (1996). Recurrence of hepatocellular carcinoma after surgery. *British Journal of Surgery*.

[11] Mathurin P, Raynard B, Dharancy S (2003). Meta-analysis: evaluation of adjuvant therapy after curative liver resection for hepatocellular carcinoma. *Alimentary Pharmacology and Therapeutics*.

[12] Ikeda K, Arase Y, Saitoh S (2000). Interferon beta prevents recurrence of hepatocellular carcinoma after complete resection or ablation of the primary tumor—a prospective randomized study of hepatitis C virus—related liver cancer. *Hepatology*.

[13] Lin SM, Lin CJ, Hsu CW, Tai DI, Sheen IS, Lin DY (2004). Prospective randomized controlled study of interferon-alpha in preventing hepatocellular carcinoma recurrence after medical ablation therapy for primary tumors. *Cancer*.

[14] Takayama T, Sekine T, Makuuchi M (2000). Adoptive immunotherapy to lower postsurgical recurrence rates of hepatocellular carcinoma: a randomised trial. *Lancet*.

[15] Taieb J, Barbare JC, Rougier P (2006). Medical treatments for hepatocellular carcinoma (HCC): what’s next?. *Annals of Oncology*.

[16] Llovet JM, Bruix J (2003). Systematic review of randomized trials for unresectable hepatocellular carcinoma: chemoembolization improves survival. *Hepatology*.

[17] Lopez PM, Villanueva A, Llovet JM (2006). Systematic review: evidence-based management of hepatocellular carcinoma—an updated analysis of randomized controlled trials. *Alimentary Pharmacology & Therapeutics*.

[18] Ruan WJ, Lai MD, Zhou JG (2006). Anticancer effects of Chinese herbal medicine, science or myth?. *Journal of Zhejiang University. Science. B.*.

[19] Treasure J (2005). Herbal medicine and cancer: an introductory overview. *Seminars in Oncology Nursing*.

[20] Luk JM, Wang X, Liu P (2007). Traditional Chinese herbal medicines for treatment of liver fibrosis and cancer: from laboratory discovery to clinical evaluation. *Liver International*.

[21] Stickel F, Schuppan D (2007). Herbal medicine in the treatment of liver diseases. *Digestive and Liver Disease*.

[22] Cohen MR (2001). Herbal and complementary and alternative medicine therapies for liver disease: a focus on Chinese traditional medicine in hepatitis C virus. *Clinics in Liver Disease*.

[23] Zhou B, Yao NL (2004). Generality of sera-pharmacologic experimental study on effect of Chinese herbal medicine on liver stellate cells. *Zhongguo Zhong Xi Yi Jie He Za Zhi*.

[24] Moher D, Cook DJ, Eastwood S, Olkin I, Rennie D, Stroup DF (1999). Improving the quality of reports of meta-analyses of randomised controlled trials: the QUOROM statement. Quality of reporting of meta-analyses. *Lancet*.

[25] Greenwald P, Milner JA, Anderson DE, McDonald SS (2002). Micronutrients in cancer chemoprevention. *Cancer and Metastasis Reviews*.

[26] Milner JA (2004). Molecular targets for bioactive food components. *Journal of Nutrition*.

[27] López-Lázaro M (2008). Anticancer and carcinogenic properties of curcumin: considerations for its clinical development as a cancer chemopreventive and chemotherapeutic agent. *Molecular Nutrition and Food Research*.

[28] Chuang SE, Kuo ML, Hsu CH (2000). Curcumin-containing diet inhibits diethylnitrosamine-induced murine hepatocarcinogenesis. *Carcinogenesis*.

[29] Chuang S-E, Cheng A-L, Lin J-K, Kuo M-L (2000). Inhibition by curcumin of diethylnitrosamine-induced hepatic hyperplasia, inflammation, cellular gene products and cell-cycle-related proteins in rats. *Food and Chemical Toxicology*.

[30] Yoysungnoen P, Wirachwong P, Changtam C, Suksamrarn A, Patumraj S (2008). Anti-cancer and anti-angiogenic effects of curcumin and tetrahydrocurcumin on implanted hepatocellular carcinoma in nude mice. *World Journal of Gastroenterology*.

[31] Yoysungnoen P, Wirachwong P, Bhattarakosol P, Niimi H, Patumraj S (2006). Effects of curcumin on tumor angiogenesis and biomarkers, COX-2 and VEGF, in hepatocellular carcinoma cell-implanted nude mice. *Clinical Hemorheology and Microcirculation*.

[32] Yoysungnoen P, Wirachwong P, Bhattarakosol P, Niimi H, Patumraj S (2005). Antiangiogenic activity of curcumin in hepatocellular carcinoma cells implanted nude mice. *Clinical Hemorheology and Microcirculation*.

[33] Ohashi Y, Tsuchiya Y, Koizumi K, Sakurai H, Saiki I (2003). Prevention of intrahepatic metastasis by curcumin in an orthotopic implantation model. *Oncology*.

[34] Lin L-I, Ke Y-F, Ko Y-C, Lin J-K (1998). Curcumin inhibits SK-Hep-1 hepatocellular carcinoma cell invasion in vitro and suppresses matrix metalloproteinase-9 secretion. *Oncology*.

[35] Lv BH, Zhang L, Zhu CC, Liu J (2007). [Inhibition of curcumin on histone deacetylase and expression promotion of P21 (WAF1/CIP1) in HepG2 cells]. *Zhongguo Zhong Yao Za Zhi*.

[36] Cao J, Liu Y, Jia L, Zhou HM, Kong Y, Yang G (2007). Curcumin induces apoptosis through mitochondrial hyperpolarization and mtDNA damage in human hepatoma G2 cells. *Free Radical Biology & Medicine*.

[37] Cao J, Jia L, Zhou H-M, Liu Y, Zhong L-F (2006). Mitochondrial and nuclear DNA damage induced by curcumin in human hepatoma G2 cells. *Toxicological Sciences*.

[38] Chan W-H, Wu H-J, Hsuuw Y-D (2005). Curcumin inhibits ROS formation and apoptosis in methylglyoxal-treated human hepatoma G2 cells. *Annals of the New York Academy of Sciences*.

[39] Ravindranath V, Chandrasekhara N (1981). In vitro studies on the intestinal absorption of curcumin in rats. *Toxicology*.

[40] Fang J-Y, Hung C-F, Chiu H-C, Wang J-J, Chan T-F (2003). Efficacy and irritancy of enhancers on the in-vitro and in-vivo percutaneous absorption of curcumin. *Journal of Pharmacy and Pharmacology*.

[41] Lançon A, Hanet N, Jannin B (2007). Resveratrol in human hepatoma HepG2 cells: metabolism and inducibility of detoxifying enzymes. *Drug Metabolism and Disposition*.

[42] Bishayee A, Dhir N (2008). Resveratrol-mediated chemoprevention of diethylnitrosamine-initiated hepatocarcinogenesis: inhibition of cell proliferation and induction of apoptosis. *Chemico-Biological Interactions*.

[43] Stervbo U, Vang O, Bonnesen C (2006). Time- and concentration-dependent effects of resveratrol in HL-60 and HepG2 cells. *Cell Proliferation*.

[44] Notas G, Nifli A-P, Kampa M, Vercauteren J, Kouroumalis E, Castanas E (2006). Resveratrol exerts its antiproliferative effect on HepG2 hepatocellular carcinoma cells, by inducing cell cycle arrest, and NOS activation. *Biochimica et Biophysica Acta*.

[45] Yan F, Tian XM, Ma XD (2006). Effects of resveratrol on growth inhibition and gap-junctional intercellular communication of HepG2 cells. *Nan Fang Yi Ke Da Xue Xue Bao*.

[46] Ma XD, Yan F, Ma AD, Wang HJ (2006). Resveratrol induces HepG2 cell apoptosis by depolarizing mitochondrial membrane. *Nan fang yi ke da xue xue bao = Journal of Southern Medical University*.

[47] Yu H, Pan C, Zhao S, Wang Z, Zhang H, Wu W (2008). Resveratrol inhibits tumor necrosis factor-*α*-mediated matrix metalloproteinase-9 expression and invasion of human hepatocellular carcinoma cells. *Biomedicine and Pharmacotherapy*.

[48] Miura D, Miura Y, Yagasaki K (2003). Hypolipidemic action of dietary resveratrol, a phytoalexin in grapes and red wine, in hepatoma-bearing rats. *Life Sciences*.

[49] Miura D, Miura Y, Yagasaki K (2004). Resveratrol inhibits hepatoma cell invasion by suppressing gene expression of hepatocyte growth factor via its reactive oxygen species-scavenging property. *Clinical & Experimental Metastasis*.

[50] Walle T, Hsieh F, DeLegge MH, Oatis JE, Walle UK (2004). High absorption but very low bioavailability of oral resveratrol in humans. *Drug Metabolism and Disposition*.

[51] Hebbar V, Shen G, Hu R (2005). Toxicogenomics of resveratrol in rat liver. *Life Sciences*.

[52] Jacobs BP, Dennehy C, Ramirez G, Sapp J, Lawrence VA (2002). Milk thistle for the treatment of liver disease: a systematic review and meta-analysis. *American Journal of Medicine*.

[53] Lieber CS, Leo MA, Cao Q, Ren C, DeCarli LM (2003). Silymarin retards the progression of alcohol-induced hepatic fibrosis in baboons. *Journal of Clinical Gastroenterology*.

[54] Wellington K, Jarvis B (2001). Silymarin: a review of its clinical properties in the management of hepaticdisorders. *BioDrugs*.

[55] Singh RP, Agarwal R (2006). Prostate cancer chemoprevention by silibinin: bench to bedside. *Molecular Carcinogenesis*.

[56] Singh RP, Agarwal R (2005). Mechanisms and preclinical efficacy of silibinin in preventing skin cancer. *European Journal of Cancer*.

[57] Varghese L, Agarwal C, Tyagi A, Singh RP, Agarwal R (2005). Silibinin efficacy against human hepatocellular carcinoma. *Clinical Cancer Research*.

[58] Lah JJ, Cui W, Hu K-Q (2007). Effects and mechanisms of silibinin on human hepatoma cell lines. *World Journal of Gastroenterology*.

[59] Momeny M, Khorramizadeh MR, Ghaffari SH, Yousefi M, Yekaninejad MS, Esmaeili R (2008). Effects of silibinin on cell growth and invasive properties of a human hepatocellular carcinoma cell line, HepG-2, through inhibition of extracellular signal-regulated kinase 1/2 phosphorylation. *European Journal of Pharmacology*.

[60] Cao E-H, Liu X-Q, Wang J-J, Xu N-F (1996). Effect of natural antioxidant Tanshinone II-A on DNA damage by lipid peroxidation in liver cells. *Free Radical Biology and Medicine*.

[61] Zhao B-L, Jiang W, Zhao Y, Hou J-W, Xin W-J (1996). Scavenging effects of Salvia miltiorrhiza on free radicals and its protection for myocardial mitochondrial membranes from ischemia-reperfusion injury. *Biochemistry and Molecular Biology International*.

[62] Wu WL, Chang WL, Chen CF (1991). Cytotoxic activities of tanshinones against human carcinoma cell lines. *American Journal of Chinese Medicine*.

[63] Ryu SY, Lee CO, Choi SU (1997). In vitro cytotoxicity of tanshinones from Salvia miltiorrhiza. *Planta Medica*.

[64] Yuan SL, Wei YQ, Wang XJ, Xiao F, Li SF, Zhang J (2004). Growth inhibition and apoptosis induction of tanshinone II-A on human hepatocellular carcinoma cells. *World Journal of Gastroenterology*.

[65] Zhong ZH, Chen WG, Liu YH, Li QX, Qiu Y (2007). Inhibition of cell growth and induction of apoptosis in human hepatoma cell line HepG2 by tanshione IIA. *Zhong Nan Da Xue Xue Bao Yi Xue Ban*.

[66] Tang Z, Tang Y, Fu L (2003). Growth inhibition and apoptosis induction in human hepatoma cells by tanshinone II A. *ournal of Huazhong University of Science and Technology Medical Science*.

[67] Wang X, Yuan S, Huang R, Song Y (1996). [An observation of the effect of tanshinone on cancer cell proliferation by Brdu and PCNA labeling]. *Hua Xi Yi Ke Da Xue Xue Bao*.

[68] Wang X, Yuan S, Wang C (1996). A preliminary study of the anti-cancer effect of tanshinone on hepatic carcinoma and its mechanism of action in mice. *Zhonghua Zhong Liu Za Zhi*.

[69] Li Q, Wang Y, Feng N, Fan Z, Sun J, Nan Y (2008). Novel polymeric nanoparticles containing tanshinone IIA for the treatment of hepatoma. *Journal of Drug Targeting*.

[70] Liu P, Mizoguchi Y, Morisawa S (1993). Effects of magnesium lithospermate B on D-galactosamine induced rat liver injury. *Zhongguo Zhong Xi Yi Jie He Za Zhi*.

[71] Matsuzaki Y, Kurokawa N, Terai S, Matsumura Y, Kobayashi N, Okita K (1996). Cell death induced by baicalein in human hepatocellular carcinoma cell lines. *Japanese Journal of Cancer Research*.

[72] Tang PM-K, Chan JY-W, Zhang D-M (2007). Pheophorbide a, an active component in scutellaria barbata, reverses P-glycoprotein-mediated multidrug resistance on a human hepatoma cell line R-HepG2. *Cancer Biology and Therapy*.

[73] Juan M, Minhu C, Yun D, Liang Q (2006). Enhancing the efficacy of photodynamic therapy by a Chinese herbal medicine for hepatocellular carcinoma. *Cancer Biology and Therapy*.

[74] Tang PM-K, Chan JY-W, Au SW-N (2006). Pheophorbide a, an active compound isolated from Scutellaria barbata, possesses photodynamic activities by inducing apoptosis in human hepatocellular carcinoma. *Cancer Biology and Therapy*.

[75] Yamashita Y, Moriyasu F, Ono S, Kimura T, Kajimura K, Someda H (1991). Photodynamic therapy using pheophorbide-a and Q-switched Nd:YAG laser on implanted human hepatocellular carcinoma. *The Japanese Society of Gastroenterology*.

[76] Oka H, Yamamoto S, Kuroki T (1995). Prospective study of chemoprevention of hepatocellular carcinoma with Sho- saiko-to (TJ-9). *Cancer*.

[77] Okita K, Li Q, Murakamio T, Takahashi M (1993). Anti-growth effects with components of Sho-saiko-to (TJ-9) on cultured human hepatoma cells. *European Journal of Cancer Prevention)*.

[78] Yano H, Mizoguchi A, Fukuda K (1994). The herbal medicine sho-saiko-to inhibits proliferation of cancer cell lines by inducing apoptosis and arrest at the G0/G1 phase. *Cancer Research*.

[79] Ha K-T, Kim J-K, Kang S-K (2004). Inhibitory effect of Sihoga-Yonggol-Moryo-Tang on matrix metalloproteinase-2 and -9 activities and invasiveness potential of hepatocellular carcinoma. *Pharmacological Research*.

[80] Watanabe S, Kitade Y, Masaki T, Nishioka M, Satoh K, Nishino H (2001). Effects of lycopene and Sho-saiko-to on hepatocarcinogenesis in a rat model of spontaneous liver cancer. *Nutrition and Cancer*.

[81] Kao ST, Yang SL, Hsieh CC, Yang MD, Wang TF, Lin JG (2000). Immunomodulation of Bu-Zhong-Yi-Qi-Tang on in vitro granulocyte colony-stimulating-factor and tumor necrosis factor-alpha production by peripheral blood mononuclear cells. *Immunopharmacology and Immunotoxicology*.

[82] Lin J-J, Jin C-N, Zheng M-L, Ouyang X-N, Zeng J-X, Dai X-H (2005). Clinical study on treatment of primary hepatocellular carcinoma by Shenqi mixture combined with microwave coagulation. *Chinese Journal of Integrative Medicine*.

[83] Li XR, Zhang D, Qi YF (2001). Experimental study on xiaoliu pingyi mixture with medicated serum in inducing apoptosis of human hepatocellular carcinoma cell line H-7402. *Zhongguo Zhong Xi Yi Jie He Za Zhi*.

[84] Chen T, Li D, Fu YL, Hu W (2008). Screening of QHF formula for effective ingredients from Chinese herbs and its anti-hepatic cell cancer effect in combination with chemotherapy. *Chinese Medical Journal*.

[85] Yin LR, Chen ZX, Zhang SJ, Sun BG, Liu YD, Huang HZ (2008). Expression of phosphatase and tensin homolog deleted on chromosome ten in liver of athymic mice with hepatocellular carcinoma and the effect of Fuzheng Jiedu Decoction. *World Journal of Gastroenterology*.

[86] Lin L-W, Sun Y, He Y-M (2004). Percutaneous intratumoral injection of traditional Chinese herbal compound medicine Star-99 in treatment of hepatocellular carcinoma of mice. *Hepatobiliary and Pancreatic Diseases International*.

[87] Tsuchiya M, Kono H, Matsuda M, Fujii H, Rusyn I (2008). Protective effect of Juzen-taiho-to on hepatocarcinogenesis is mediated through the inhibition of Kupffer cell-induced oxidative stress. *International Journal of Cancer*.

[88] Yamamoto S, Oka H, Kanno T, Mizoguchi Y, Kobayashi K (1989). Controlled prospective trial to evaluate Syosakiko-to in preventing hepatocellular carcinoma in patients with cirrhosis of the liver. *Gan To Kagaku Ryoho*.

[89] Meng M-B, Cui Y-L, Guan Y-S (2008). Traditional Chinese medicine plus transcatheter arterial chemoembolization for unresectable hepatocellular carcinoma. *Journal of Alternative and Complementary Medicine*.

[90] Shu X, McCulloch M, Xiao H, Broffman M, Gao J (2005). Chinese herbal medicine and chemotherapy in the treatment of hepatocellular carcinoma: a meta-analysis of randomized controlled trials. *Integrative Cancer Therapies*.

[91] Chen ZX, Zhang SJ, Hu HT, Sun BG, Yin LR (2007). [Clinical study of method of strengthening body resistance and disintoxication disintoxication in patients with HCC of post-TACE]. *Zhongguo Zhong Yao Za Zhi*.

[92] Chao JC-J, Chiang S-W, Wang C-C, Tsai Y-H, Wu M-S (2006). Hot water-extracted Lycium barbarum and Rehmannia glutinosa inhibit proliferation and induce apoptosis of hepatocellular carcinoma cells. *World Journal of Gastroenterology*.

[93] Chang J-S, Chiang L-C, Hsu F-F, Lin C-C (2004). Chemoprevention against hepatocellular carcinoma of Cornus officinalis in vitro. *American Journal of Chinese Medicine*.

[94] Yamashiki M, Nishimura A, Nomoto M, Suzuki H, Kosaka Y (1996). Herbal medicine ’Sho-saiko-to’ induces tumour necrosis factor-*α* and granulocyte colony-stimulating factor in vitro in peripheral blood mononuclear cells of patients with hepatocellular carcinoma. *Journal of Gastroenterology and Hepatology*.

[95] Qian J, Zheng ZS, Wu HP (2005). The application of Bletilla striata in the interventional therapy of hepatocellular carcinoma: a comparative study using ACI rats. *Zhonghua Gan Zang Bing Za Zhi*.

[96] Shiota G, Maeta Y, Mukoyama T (2002). Effects of Sho-saiko-to on hepatocarcinogenesis and 8-hydroxy-2′-deoxyguanosine formation. *Hepatology*.

